# Applying Machine Learning Models with An Ensemble Approach for Accurate Real-Time Influenza Forecasting in Taiwan: Development and Validation Study

**DOI:** 10.2196/15394

**Published:** 2020-08-05

**Authors:** Hao-Yuan Cheng, Yu-Chun Wu, Min-Hau Lin, Yu-Lun Liu, Yue-Yang Tsai, Jo-Hua Wu, Ke-Han Pan, Chih-Jung Ke, Chiu-Mei Chen, Ding-Ping Liu, I-Feng Lin, Jen-Hsiang Chuang

**Affiliations:** 1 Taiwan Centers for Disease Control Taipei Taiwan; 2 Value Lab Acer Inc. Taipei Taiwan; 3 National Taipei University of Nursing and Health Sciences Taipei Taiwan; 4 Institute of Public Health National Yang-Ming University Taipei Taiwan

**Keywords:** influenza, Influenza-like illness, forecasting, machine learning, artificial intelligence, epidemic forecasting, surveillance

## Abstract

**Background:**

Changeful seasonal influenza activity in subtropical areas such as Taiwan causes problems in epidemic preparedness. The Taiwan Centers for Disease Control has maintained real-time national influenza surveillance systems since 2004. Except for timely monitoring, epidemic forecasting using the national influenza surveillance data can provide pivotal information for public health response.

**Objective:**

We aimed to develop predictive models using machine learning to provide real-time influenza-like illness forecasts.

**Methods:**

Using surveillance data of influenza-like illness visits from emergency departments (from the Real-Time Outbreak and Disease Surveillance System), outpatient departments (from the National Health Insurance database), and the records of patients with severe influenza with complications (from the National Notifiable Disease Surveillance System), we developed 4 machine learning models (autoregressive integrated moving average, random forest, support vector regression, and extreme gradient boosting) to produce weekly influenza-like illness predictions for a given week and 3 subsequent weeks. We established a framework of the machine learning models and used an ensemble approach called stacking to integrate these predictions. We trained the models using historical data from 2008-2014. We evaluated their predictive ability during 2015-2017 for each of the 4-week time periods using Pearson correlation, mean absolute percentage error (MAPE), and hit rate of trend prediction. A dashboard website was built to visualize the forecasts, and the results of real-world implementation of this forecasting framework in 2018 were evaluated using the same metrics.

**Results:**

All models could accurately predict the timing and magnitudes of the seasonal peaks in the then-current week (nowcast) (ρ=0.802-0.965; MAPE: 5.2%-9.2%; hit rate: 0.577-0.756), 1-week (ρ=0.803-0.918; MAPE: 8.3%-11.8%; hit rate: 0.643-0.747), 2-week (ρ=0.783-0.867; MAPE: 10.1%-15.3%; hit rate: 0.669-0.734), and 3-week forecasts (ρ=0.676-0.801; MAPE: 12.0%-18.9%; hit rate: 0.643-0.786), especially the ensemble model. In real-world implementation in 2018, the forecasting performance was still accurate in nowcasts (ρ=0.875-0.969; MAPE: 5.3%-8.0%; hit rate: 0.582-0.782) and remained satisfactory in 3-week forecasts (ρ=0.721-0.908; MAPE: 7.6%-13.5%; hit rate: 0.596-0.904).

**Conclusions:**

This machine learning and ensemble approach can make accurate, real-time influenza-like illness forecasts for a 4-week period, and thus, facilitate decision making.

## Introduction

Seasonal influenza is one of the most prevalent infectious diseases in Taiwan, accounting for millions of cases, over tens of thousands of patient hospitalizations, and hundreds of deaths annually [1-4]. In Taiwan, the seasonal influenza epidemic typically begins in winter and continues to the end of the year until the spring of the next year [2]. However, the changeful influenza activity in subtropical areas like Taiwan sometimes causes problems in epidemic preparedness. For instance, in Taiwan, the 2015-2016 influenza epidemic, with H1N1 as the main circulating strain, was the biggest since the 2009 novel H1N1 outbreak. Nevertheless, H1N1 influenza activity was unexpectedly low in the following 2016-2017 influenza season, whereas H3N2 influenza activity peaked unpredictably in the summer season in 2017 and caused a severe epidemic [2,3].

Since 2004, to monitor changes in influenza activity, the Taiwan Centers for Disease Control (Taiwan CDC) has established real-time national influenza surveillance systems for influenza-like illness visits to hospitals and clinics [5,6]. The surveillance systems have minimal time lag in data collection; therefore, public-health professionals can immediately adjust their response almost in real time. The decision-making process, however, remains based only on past data (despite the short time lag). Influenza epidemic forecasting for upcoming weeks or months can provide more information for policymaking and is relevant for preparedness [7]. The ability to provide a short-term forecast in terms of epidemic magnitude is particularly vital for emergency departments during a long weekend or the Lunar New Year in Taiwan (eg, the Lunar New Year comprised 9 vacation days in 2019), during which time, influenza-like illness visits at emergency departments considerably increase (since outpatient services are closed), and sometimes patients crowd the emergency departments. In this situation, reliable forecasts are required to determine the surging capacity.

Many research teams have worked on influenza forecasting for a long time. Among the models used by researchers, the autoregressive integrated moving average (ARIMA) model is a methodology that is often chosen for seasonal influenza forecasts because of its advantage in dealing with time-series data [8,9], its satisfactory performance using data that are time dependent for short-term projection, and its widespread use in other health-related forecasting tasks [9-14]. Decision tree–based machine learning algorithms such as random forest and extreme gradient boosting also have their strengths in predictive analysis and forecasting, which has been shown in data science competitions such as Kaggle [15], influenza outbreaks [14], and foodborne disease trends [16]. A study in Canada [17] showed random forest models had better performance predicting influenza A virus frequency than that of ARIMA and generalized linear autoregressive integrated moving average models. Unlike ARIMA, random forest and extreme gradient boosting, as ensemble weak prediction models, have better performance dealing with high-dimension data [18], while support vector regression’s strength is finding an optimal hyperplane with a nonlinear boundary [19,20]. Previous research has also demonstrated a successful combination of linear regression with nonlinear predictor, random forest, support vector regression, and extreme gradient boosting to predict dengue fever outbreak in the United States [21].

Instead of traditional surveillance data, researchers have also attempted using nontraditional data sources, such as Google Flu Trends and Flu Near You, to improve their forecasts since 2008 [22,23]. These data served as surrogate indicators or supplement data for influenza-like illness activity. Lasso regression, random forest, extreme gradient boosting, and support vector regression have been widely implemented to aggregate these data from Google search, Google trend, Wikipedia, and social media (such as Twitter and Baidu) in influenza forecasting [24-27]. The performance of elastic net and support vector regression was considered to be comparable in a study [26] which used the Baidu index as a predictor and predicted the number of influenza cases in China by support vector regression, and in a study [28] in France which used electronic health record data with historical epidemiology information for influenza-like illness incidence rate predictions.

On the other hand, researchers began to explore the possibility of simultaneously using multiple models or data sources to find an ensemble approach to produce more robust forecasts by combining the results of different forecasting models [11,29-32]. For seasonal influenza forecasting in the US, the empirical Bayes method has been used to integrate the forecasts from linear models using multiple data sources as predictors [29]. Kandula et al [32] also evaluated the performance of the susceptible-exposed-infectious-recovered-susceptible model, Bayesian weighted outbreaks, k-nearest neighbor, and a superensemble method when combining distinct forecast methods to predict influenza outbreaks in the United States. A meta-ensemble of statistical and mechanistic methods has shown better accuracy than individual methods [31,32].

Compared to internet data, which might easily be influenced by search engine marketing, the surveillance database in Taiwan can provide much more comprehensive data with a small time lag. These data sources are also easier to be maintained and reliable for a long-term decision-making system. Therefore, using the surveillance data, we aimed to develop a practical framework consisting of an ensemble model with machine learning models to combine the advantages of different forecasting models for real-time influenza-like illness predictions, and facilitate influenza preparedness.

## Methods

### Data Source

The data we used to train and validate the machine learning algorithm included weekly data from the Real-Time Outbreak and Disease Surveillance System, the National Health Insurance Database, and the National Notifiable Disease Surveillance System [5,6]. The details and characteristics of the data sets are described in Multimedia Appendix 1. Other data used include the number of national holidays in each week, regular weekends, and long weekends. We used surveillance data from 2008-2017 to establish the framework of the forecasting models.

### Forecasting Targets and the Renewal of the Surveillance Data and Models

**Figure 1:**
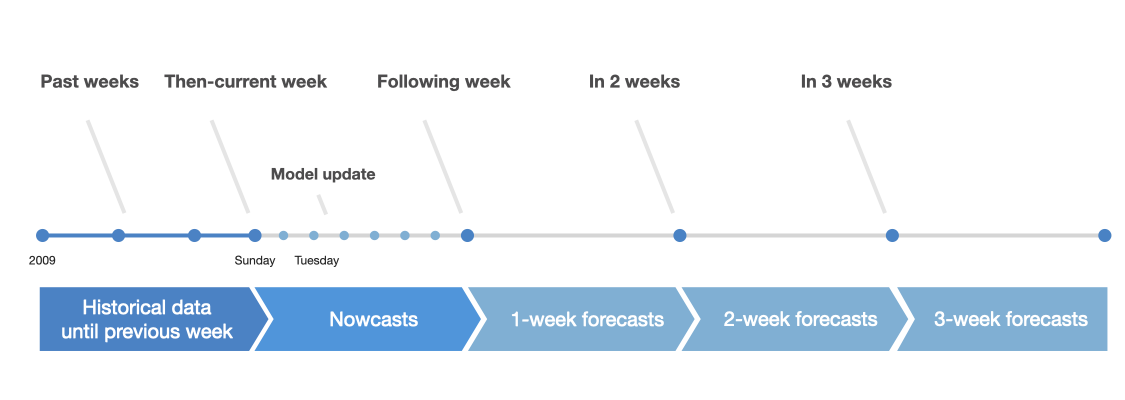
Timeline of historical data used for model training and forecasting periods.

The forecasting targets in our study were short-term forecasts—weekly number of influenza-like illness visits for the 4-week period after the most recent surveillance data. Real-Time Outbreak and Disease Surveillance System, National Health Insurance, and National Notifiable Disease Surveillance System databases were updated daily. Because of the potential delay in data entry by hospitals, all the models were automatically retrained and updated every Tuesday night using data up until the end of the previous week. The updated models would then produce the predictions of influenza-like illness visits for 4 weeks from that time point (the end of the previous week). Therefore, the initial forecast actually predicts the number of influenza-like illness visits in the then-current week (*nowcast*), whereas the *1-week*, *2-week*, and *3-week forecasts* represent the weekly predictions for each of the subsequent 3 weeks (Figure 1).

### Machine Learning Algorithms

Four machine learning algorithms—ARIMA, random forest, support vector regression, and extreme gradient boosting—were used to produce weekly influenza-like illness predictions for a 4-week period. We chose these algorithms, each with different characteristics and strengths, so that the forecasting task could benefit from the diversity of the machine learning algorithms.

To summarize the forecasts of the 4 different machine learning algorithms, we adopted the ensemble method called stacking [31,33] An ensemble model was trained using another support vector regression algorithm with a linear kernel that optimized the best regression between the observed number of influenza-like illness visits and the 4-week forecasts. A previous study [32] adopted a Bayesian model, which requires the prior distribution estimation to produce the ensemble forecast. We chose a support vector regression with a linear kernel model because it can produce a weighted-average forecast from 4 individual models without considering data distribution. By using the stacking method, the forecasts of different algorithms are automatically weighted and combined to produce the ensemble forecasts. The linear kernel was chosen because of the forecasting and efficient computing performance it showed in the training process. The hyperparameter tuning mechanism, described in the section that follows, was used to evaluate the performance of the ensemble model from the first week of 2015 to the 40th week of 2017.

### Feature Selection, Engineering, and Model Tuning

The initial features were selected after discussions with experts of Taiwan CDC. The number of past influenza-like illness visits in the 8 previous weeks (from the Real-Time Outbreak and Disease Surveillance System and National Health Insurance database) and the length of national holidays in a week were the basic features. We also included essential holidays, such as the Lunar New Year, in the feature set because it was believed to have a significant influence on influenza-like illness visits, especially in emergency departments. Our feature engineering work included moving average, moving difference with varying time lags, and the proportion of influenza-like illness visits to total medical visits (Multimedia Appendix 2).

**Figure 2:**
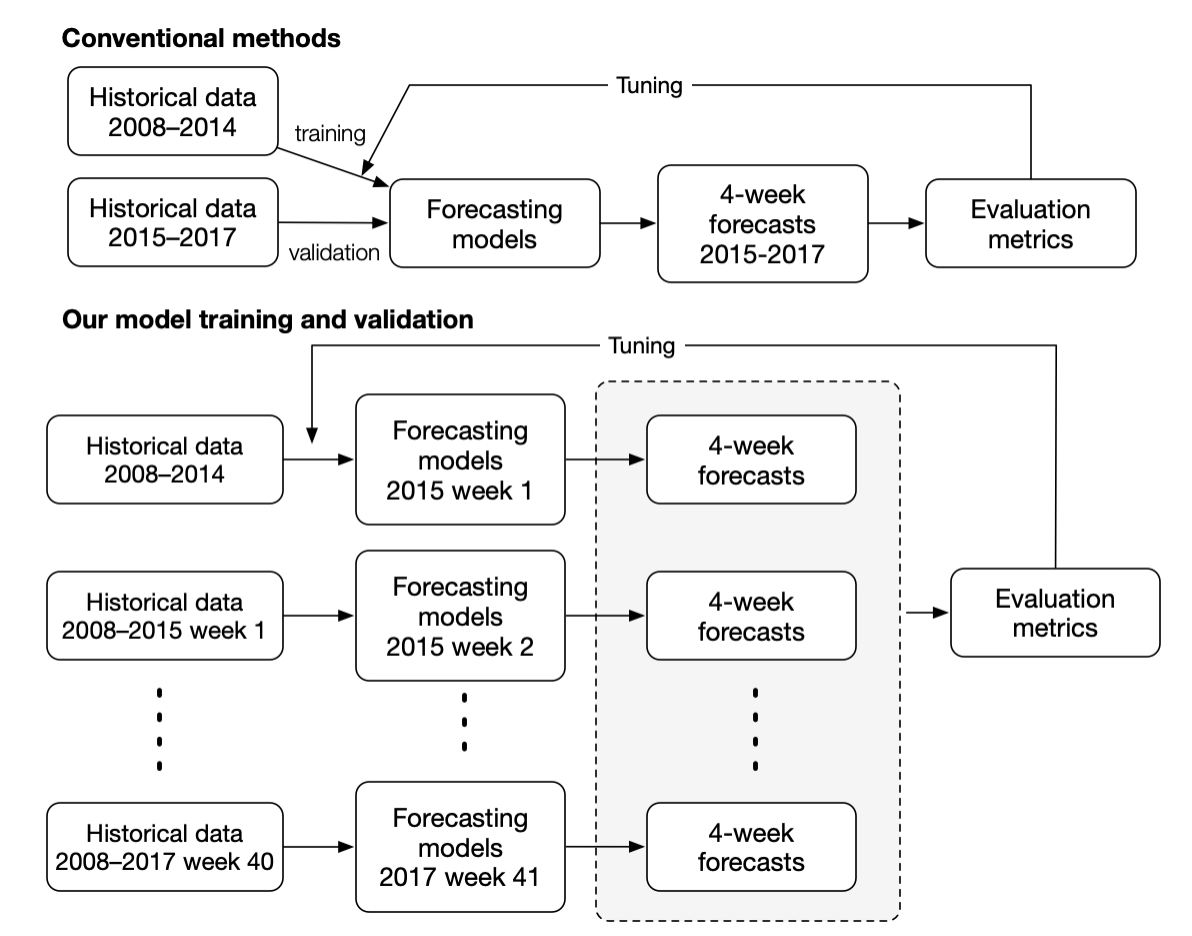
The framework of feature selection and model tuning for our model training and validation compared to the conventional method.

We chose naïve (heuristic) mechanisms, instead of the conventional methods, for feature selection. We used surveillance data from 2008-2017 for feature selection and model tuning. We evaluated the overall forecasting performance of the algorithms during the first week of 2015 to the 40th week of 2017 by comparing the forecasts to observed historical data in the same period. Using this framework, we dynamically retrained each model from zero every week to incorporate newly collected data (Figure 2):

1. For an individual week *T* from the first week of 2015, the training data set included the weekly influenza-like illness data from week 1 in 2008 to week *T*. 

2. Given a set of features and fixed hyperparameter value, the model *f* (·|*T*, *h*), forecasting the *h* weeks ahead, was trained using the training data set at week *T* .

3. The number of influenza-like illness visits in week *T* + *h* was forecasted by the trained *f* (·|*T*, *h*).

4. The forecasted number was compared to the observed number of influenza-like illness visits in week *T* + *h* using the evaluation metrics.

5. For each week from the first week of 2015 to the 40th week of 2017, we repeated Step 1 to Step 4 and calculated the evaluation metrics for specific feature sets and hyperparameters. Then we selected the feature set and hyperparameters that performed best in evaluation metrics.

If we only used k-fold cross-validation during model training, look-ahead bias might have occurred when using time-series data with potential autocorrelation. The advantage of this framework avoided look-ahead bias and made use of all historical data before the week *T* to train the models in forecasting the weekly influenza-like illness visits of the week *T* + *h* at the week *T*. 

### Evaluation Metrics

The metrics we used to evaluate the model performance included Pearson correlation (ρ), root mean squared error (RMSE), mean absolute percentage error (MAPE), and hit rate (Multimedia Appendix 3). Lower MAPE, lower RMSE, higher hit rate, and higher correlation indicated better forecasting performance.

### Software and Visualization

We used data munging and feature engineering (dplyr), the time-series model, ARIMA (forecast) , random forest model (randomForest), support vector regression (e1071), and extreme gradient boosting model (xgboost) packages in R (version 3.4.4) on Ubuntu (version 14.0.4). The functions and hyperparameters that were used are listed in Multimedia Appendix 4. A visualization dashboard website was designed to display and compare the predictions of the 5 models (using D3.js and several JavaScript frameworks) [34].

## Results

### Real-Time Estimates (Nowcast)

**Figure 3:**
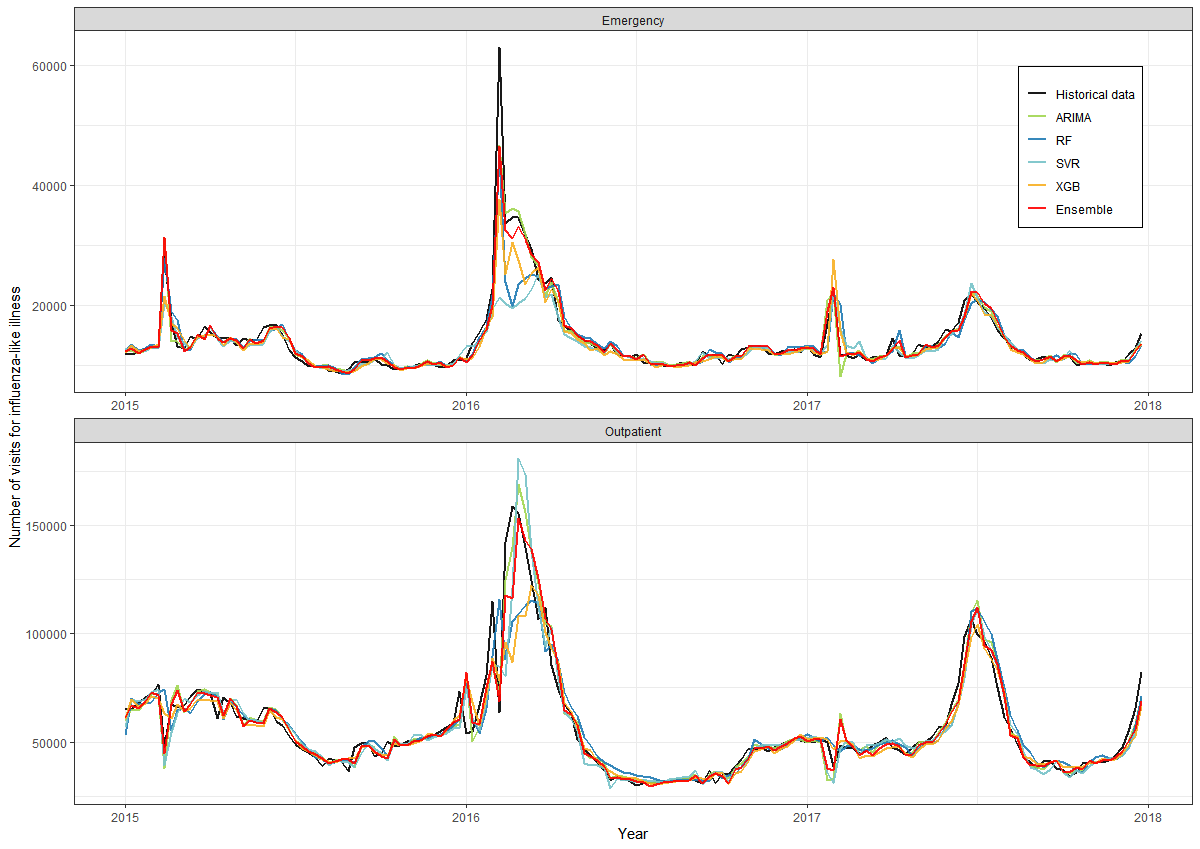
Nowcasts (current week predictions) of the influenza-like illness visits in outpatient and emergency departments by the 5 machine learning models (colored lines) compared with the observed historical data (black line), 2015-2017. ARIMA: autoregressive integrated moving average; ILI: influenza-like illness; RF: random forest; SVR: support vector regression; XGB: extreme gradient boosting.

The visualized comparison of the estimated epidemic curve in nowcasts and the observed number of influenza-like illness visits showed that all the models, especially the ensemble model, could predict the time and magnitude of the peaks of the influenza epidemic throughout the influenza season from 2015-2017, such as the peaks of the Lunar New Year vacation for each year and the peak of the summer flu in 2017 (Figure 3). All models could appropriately fit the epidemic curve of the outpatient (ρ=0.891-0.962) and emergency (ρ=0.802-0.967) departments (Table 1).

For 2015-2017, the nowcast prediction by the 4 machine learning models exhibited good accuracy (MAPE as low as 5.2%); however, the ensemble model (outpatient: ρ=0.956, MAPE 6.0%, hit rate 0.756; emergency: ρ= 0.967, MAPE 5.2%, hit rate 0.705) outperformed individual models.

**Table 1:** The evaluation metrics of the 5 machine learning models for then-current week forecasts (nowcast), 1-week forecasts, 2-week forecasts, and 3-week forecasts for 2015 to 2017 data.

Time period		Outpatient influenza-like illness visits				Emergency influenza-like illness visits			
	Model	RMSE^a^	MAPE^b^, %	Hit rate	Pearson correlation coefficient	RMSE	MAPE, %	Hit rate	Pearson correlation coefficient
**Nowcast (current week)**									
	ARIMA^c^	6621.9	6.5	0.744	0.962	1689.8	5.2	0.718	0.965
	RF^d^	10773.1	9.2	0.577	0.891	2707.8	7.6	0.609	0.922
	SVR^e^	9265.0	7.7	0.686	0.923	4189.8	7.1	0.686	0.802
	XGB^f^	10063.6	7.3	0.635	0.915	2696.0	6.6	0.667	0.935
	Ensemble	6903.5	6.0	0.756	0.956	1696.3	5.2	0.705	0.967
**1-week**									
	ARIMA	11165.2	11.5	0.695	0.892	2562.1	8.3	0.747	0.918
	RF	12256.6	11.7	0.701	0.855	3430.0	11.8	0.643	0.842
	SVR	11573.6	10.7	0.740	0.874	4351.8	9.4	0.701	0.803
	XGB	13604.7	11.0	0.695	0.836	3866.7	9.7	0.688	0.842
	Ensemble	11752.8	10.1	0.721	0.874	2831.6	8.3	0.708	0.901
**2-week**									
	ARIMA	15471.8	15.3	0.695	0.792	3206.2	10.1	0.727	0.867
	RF	13464.5	13.8	0.721	0.823	3639.8	13.7	0.669	0.816
	SVR	13972.9	13.7	0.708	0.808	4562.1	10.8	0.734	0.783
	XGB	15317.7	13.6	0.727	0.785	4235.0	11.3	0.708	0.817
	Ensemble	13758.1	12.0	0.727	0.823	3467.9	10.4	0.727	0.860
**3-week**									
	ARIMA	19338.3	18.9	0.669	0.676	3836.8	12.0	0.688	0.801
	RF	14310.9	14.9	0.753	0.796	3949.8	15.0	0.708	0.777
	SVR	16004.3	15.9	0.786	0.743	4903.4	12.1	0.675	0.731
	XGB	16888.8	15.7	0.708	0.723	4823.2	13.5	0.734	0.686
	Ensemble	15193.6	13.3	0.773	0.780	3937.2	13.1	0.643	0.797

^a^RMSE: root mean squared error.

^b^MAPE: mean absolute percentage error.

^c^ARIMA: autoregressive integrated moving average.

^d^RF: random forest.

^e^SVR: support vector regression.

^f^XGB: extreme gradient boosting.

### Forecasts for the Following 3 Weeks

**Figure 4:**
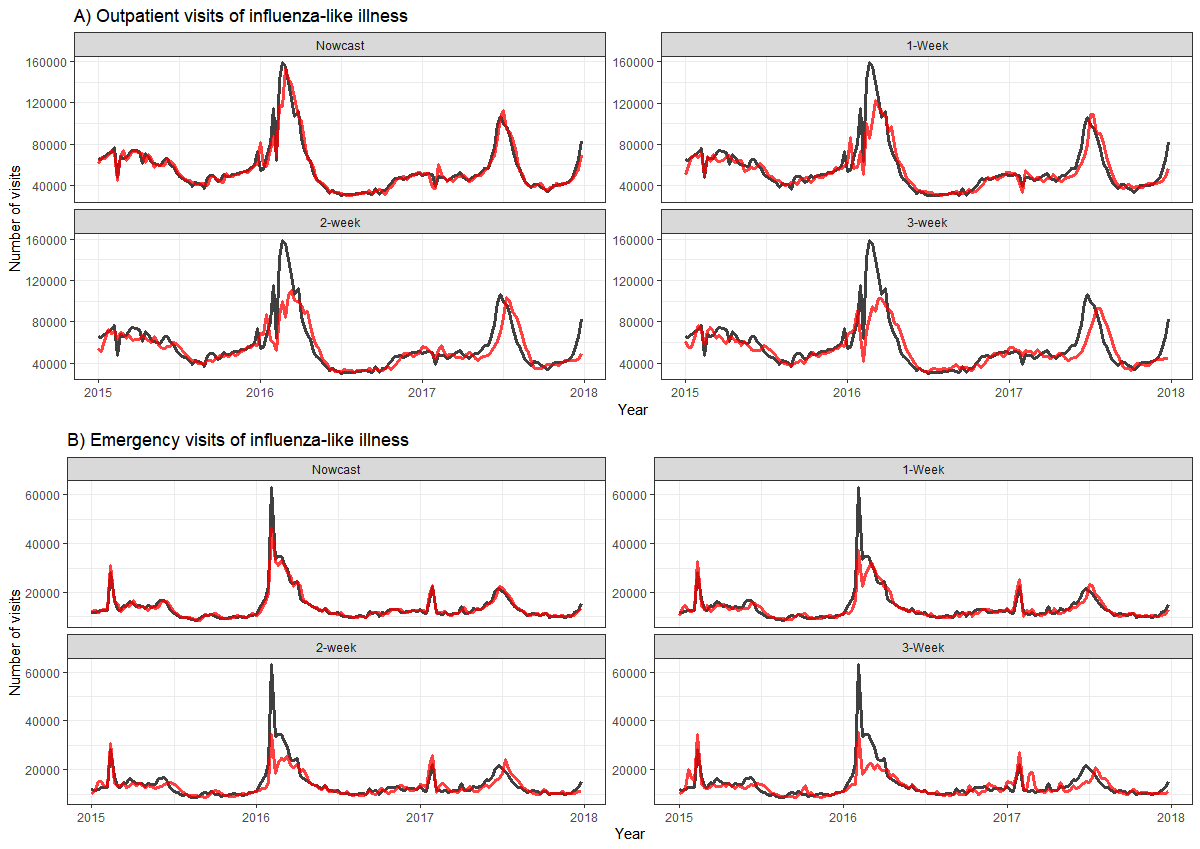
Forecasts using the ensemble model (red) and the observed (black) number for influenza-like illness visits in (A) outpatient and (B) emergency departments, 2015-2017.

The forecasts for the following 3 weeks using our ensemble model exhibited satisfactory performance for predicting the epidemic trend and successfully captured the epidemic peaks. Still, there were some time lags in peak prediction in the 1-, 2-, and 3-week forecasts (Figure 4). The accuracy slightly decreased with an increase in the forecast time horizons as well (MAPE: 8.3%-18.9%; hit rate: 0.643-0.786 in the 1-week, 2-week, and 3-week forecasts) (Table 1). Although the ARIMA model had the highest accuracy and hit rate in nowcasts, the random forest and support vector regression models performed better in the forecasts of the subsequent 2 and 3 weeks, particularly in terms of outpatient influenza-like illness visits.

### Real-World Application in 2018

**Figure 5:**
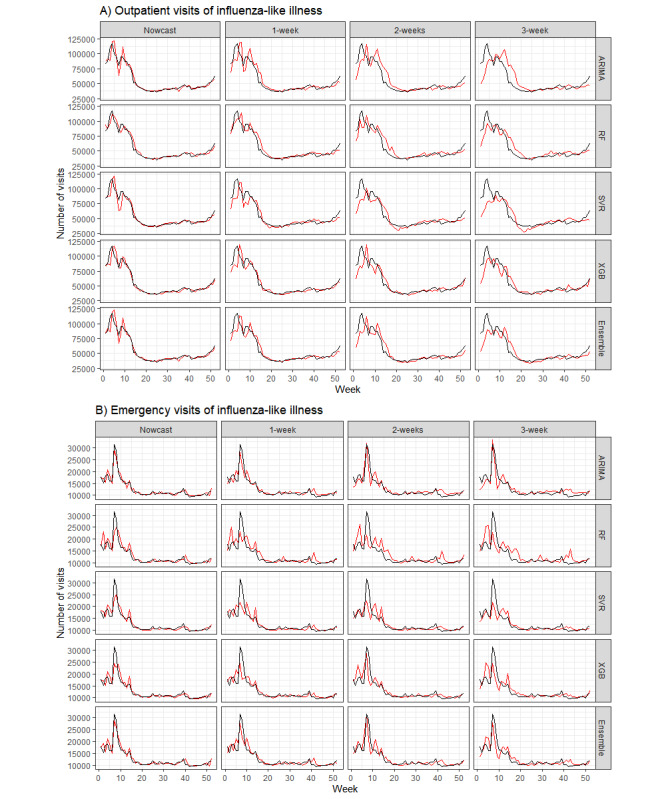
Forecasts of the 5 machine learning models (red) and the observed number (black) of influenza-like illness visits in (A) outpatient and (B) emergency departments, 2018. ARIMA: autoregressive integrated moving average; MAPE: mean absolute percentage error; RF: random forest; RMSE: root mean squared error; SVR: support vector regression; XGB: extreme gradient boosting.

We started using the framework with the 5 models in Taiwan CDC in early 2018. Since 2018, the nowcasts of our models has exhibited good accuracy (outpatient MAPE: 5.3%-5.8%; emergency MAPE: 5.7%-8.0%). Moreover, the 3-week forecasts maintained comparable accuracy to one another (outpatient MAPE: 8.8%-13.5%; emergency MAPE: 8.8%-13.5%; Table 2 and Multimedia Appendix 5). Hit rates of the nowcasts were 0.600-0.727 in outpatient and 0.582-0.782 in emergency department and remained at a high level in the 3-week forecasts (0.787-0.908 and 0.596-0.788 in outpatient and emergency department, respectively). All the models could approximately detect the declining trend when the magnitude of the epidemic had already reached a peak (Figure 5). The random forest and extreme gradient boosting model better identified the increasing trend during the early stage of the epidemic. 

**Table 2:** Evaluation metrics of the 5 machine learning models for the current week forecasts (nowcast), 1-week forecasts, 2-week forecasts, and 3-week forecasts in 2018.

			Outpatient influenza-like illness visits					Emergency influenza-like illness visits			
	Model	RMSE^a^		MAPE^b^, %	Hit rate	Pearson correlation coefficient	RMSE		MAPE, %	Hit rate	Pearson correlation coefficient
**Nowcast (current week)**											
	ARIMA^c^	6422.8		5.5	0.727	0.958	1125.3		5.7	0.782	0.965
	RF^d^	6472.1		5.4	0.709	0.957	1305.8		6.2	0.709	0.952
	SVR^e^	5343.2		5.8	0.655	0.969	2079.6		8.0	0.582	0.875
	XGB^f^	7384.6		5.8	0.691	0.943	1643.6		6.2	0.673	0.923
	Ensemble	6170.7		5.3	0.600	0.962	1751.0		6.5	0.727	0.912
**1-week**											
	ARIMA	9874.7		9.0	0.741	0.897	1707.6		7.8	0.704	0.919
	RF	8644.1		7.2	0.833	0.921	1861.2		7.9	0.759	0.899
	SVR	7330.1		7.9	0.778	0.942	2643.7		10.6	0.759	0.798
	XGB	9738.7		9.0	0.741	0.903	2353.3		8.3	0.685	0.836
	Ensemble	9156.8		7.5	0.796	0.911	2363.0		8.4	0.722	0.832
**2-week**											
	ARIMA	11630.6		11.8	0.811	0.851	1922.2		8.6	0.755	0.893
	RF	10082.3		9.4	0.811	0.893	1975.5		7.7	0.736	0.888
	SVR	9292.7		10.1	0.830	0.905	3031.0		12.8	0.566	0.730
	XGB	11262.8		11.3	0.755	0.873	2371.6		7.9	0.698	0.843
	Ensemble	10078.8		8.6	0.774	0.889	2389.4		8.6	0.755	0.835
**3-week**											
	ARIMA	13656.7		13.5	0.865	0.787	1875.4		9.5	0.788	0.898
	RF	10258.0		10.1	0.769	0.892	2041.9		8.8	0.692	0.877
	SVR	9439.7		10.9	0.885	0.904	3106.0		13.5	0.596	0.721
	XGB	12789.3		13.0	0.788	0.830	2259.6		7.6	0.692	0.890
	Ensemble	9160.9		8.8	0.904	0.908	2478.4		9.6	0.769	0.814

^a^RMSE: root mean squared error.

^b^MAPE: mean absolute percentage error.

^c^ARIMA: autoregressive integrated moving average.

^d^RF: random forest.

^e^SVR: support vector regression.

^f^XGB: extreme gradient boosting.

### Visualization Dashboard of Forecasts

To easily compare the predictions of the 5 models, we created a visualization dashboard website to display the projections concurrently (Multimedia Appendix 6). We also provided the MAPEs and hit rates of all the models that were calculated using the recent 4-, 8-, and 52-week data. In this manner, policy makers could also consider accuracy when evaluating predictions.

## Discussion

### Principal Results

By using the influenza surveillance data from Taiwan CDC, we established a forecasting model framework that comprises 4 machine learning models and one ensemble model. Our ensemble approach and the framework of model training could provide highly precise forecasts of weekly influenza-like illness visits for a 4-week period. Then-current week forecasts (nowcasts) were the most accurate with MAPE as low as approximately 5% and hit rates of approximately 0.75. Because of the satisfactory hit rate, the change in the influenza-like illness visits in the forecasts for the 4-week period could be regarded as the estimated temporal trend forecasts of a future epidemic as well.

A comparison with models developed in other countries or areas revealed that our models could provide better accuracy with a very low MAPE, which was less than 10% in nowcasts and remained below 20% in the 4-week period forecasts. These results outperform previously-reported models with MAPE mostly greater than 15%-20% [11,25,35,36], suggesting that our models can provide promising predictivity of short-term forecasts on the epidemic magnitude; however, it is difficult to directly compare the performance among different algorithms developed in varying clinical settings and with varying data quality. As for short-term forecasting of the epidemic magnitude in Taiwan, an MAPE less than 10%, especially during the peak time, would be helpful when policymakers need to evaluate the required surging capacity. Because the weekly change of influenza-like illness visits is usually less than 10%-15% in Taiwan, an MAPE greater than 15%-20% might not reliably catch the shift in the epidemic.

The high accuracy of our models might be attributed to the comprehensive data set that we used [5]. The high coverage and good representativeness of the Real-Time Outbreak and Disease Surveillance System and National Health Insurance database allowed the forecasts to more accurately reflect the trends and magnitude of influenza-like illness without being affected by the bias that would have been caused by incompletely sampled data. Conversely, previous models in the US and other countries mostly relied on the sentinel surveillance system such as the influenza-like illness net in the US, which was mainly composed of volunteered sentinel clinics and had problems pertaining to completeness and representativeness [37-40]; the predictivity of the models might have been significantly impaired when they were trained using imprecise historical data. Researchers usually develop an algorithm using a specific period of historical data and then use the trained algorithm with newly-collected data to forecast; therefore, forecasting performance can become worse and worse over time and require periodic adjustment.

In contrast, with our method, models can be retrained every week using updated data. In this way, the algorithms learn from the updated data and maintain satisfactory performance even after being used by the Taiwan CDC for more than one year. In addition, our ensemble model was adapted from the stacking method and could summarize the forecasting outputs from the 4 basic machine learning algorithms with appropriate weighting [31-33]. The aim of our ensemble model was not to build the most accurate forecasting model for any given time. Since the 4 models select features independently from our data sources and had different forecasting performance in real-world applications, for example, ARIMA was usually a lagging indicator of the peak in the influenza season, while random forest and extreme gradient boosting predicted the peak better but tended to underestimate the magnitudes at the peak; therefore, by combining the forecasts from ARIMA and extreme gradient boosting model, the ensemble approach could overcome the disadvantages of each individual model and generate the most robust forecasts with stable performance.

In addition to completeness and representativeness, the Real-Time Outbreak and Disease Surveillance System and National Health Insurance database provided excellent timeliness for our forecasting models. Thanks to the nearly real-time data exchange of the Real-Time Outbreak and Disease Surveillance System and National Health Insurance database with a time lag of, at the most, 1-2 days [5], we could use influenza-like illness data from the previous week at the beginning the week and generate forecasts for a 4-week period that started every Tuesday, for any given week. Because of the delay in the collection of surveillance data, models developed in other countries usually acquire data with at least 1-2 weeks of delay. Thus, their 1-week forecast generated using historical data up until 2 weeks prior is actually the prediction for the previous week [11,25,40-42]. Compared to those models [43], the aforementioned short time delay made our forecast model, which can generate the forecast of a given week (nowcast), a real *real-time forecasting* model. This information can be of great help to the authorities for decision making concerning epidemic preparedness and interventions.

In order to resolve the timeliness problem of the influenza-like illness surveillance data, researchers have attempted to explore the use of social media data (such as Twitter and Facebook) or internet search data (such as Google search and Google Flu Trends) to develop forecasting models because these data can be collected in almost real-time [11,22,26,36,41,44]. However, the method of data collection, quality of social media data, and accuracy of the models still posed problems [22,26,36,41]. In our framework, we did not include social media data because of the following reasons. First, ideal sources of social media information have not yet been established in Taiwan. The largest social media website in Taiwan is Facebook. Still, it is rarely used in social media surveillance because of the hindrances in collecting personal posts from individual profiles (personal walls). A microblog such as Twitter is less prevalent in Taiwan netizens. Second, as for web search data, the Taiwan CDC conducts a regular weekly press release and usually causes a higher amount of search for the related terms on the day of the press release. For example, the searches for influenza significantly increase when an influenza-related news article is released. Therefore, it is difficult to determine whether the increase in the number of web searches, epidemic-related news, or social media discussions is due to an increase influenza-like illness visits or the effect on the media of the official press release. Conversely, access to medical service in Taiwan is easy, and our surveillance systems, such as the Real-Time Outbreak and Disease Surveillance System and the National Health Insurance database, have already collected highly comprehensive data. Thus, we do not need to rely upon the use of social media as a supplementary data source for disease forecasting, especially for influenza-like illness.

For the models based on traditional frequency statistics such as ARIMA, it is relatively easy to produce a 95% confidence interval, but it is not similarly easy for random forest, support vector regression, and extreme gradient boosting models. Although some literature discussed how to generate prediction intervals for machine learning models like random forest, it is not practical to display 5 intervals on one chart simultaneously. Too much information only confuses the user and makes it difficult to interpret the trend from the 5 forecasts. As we introduced 4 forecasting models based on different algorithms, they already provide and demonstrate the variations in forecasts. When we combine these forecasts with the most robust forecasts from the ensemble model, decision makers can easily get an impression of the forecast without ignoring the potential outlier at one time. Thus, this framework can provide similar information to that provided by confidence intervals.

The models used were machine learning models, which were different from traditional mechanistic models and those such as the susceptible-infectious-recovered model [45,46]. The susceptible-infectious-recovered model considers the dynamics of infectious disease and other biological components. For example, a researcher could create a compartment to simulate the interaction dynamics between infected and immunized people to estimate the effects of vaccination. However, these models are usually built on the basis of historical data and are useful in evaluating the relationship between the different compartments. This characteristic makes such a model better for assessing the effectiveness of vaccination or other interventions on disease transmission, but poor in making future prediction since it is difficult to extrapolate the results because of unknown data at forecasting [43]. For example, when building a susceptible-infectious-recovered-V model, including the compartment V as vaccinated, we need to enter the possible number of vaccinated people in the near future if we want to use this model for forecasting.

### Limitations

There are some limitations to our forecasting models. First, the predictivity of our models decreased with longer time horizons, and the best hit rate was only approximately 0.75, suggesting that our models are better at predicting the epidemic magnitude but not the trend. However, we could calibrate the forecasts by learning from the experience of the real-world application. For example, compared with traditional time-series models, such as the ARIMA model, we found that the random forest and support vector regression models may better predict the epidemic dynamics, when the models were applied in 2018. By combining the forecasts and human judgment, the decision-making process for future epidemics can be further ameliorated. Second, using other new deep learning algorithms, especially those with promising performance in time-series forecasting tasks, such as a recurrent neural network and long short-term memory networks, may help to improve the forecasting accuracy. Unlike sequential learning, we retrained the models from zero with mostly updated data every week to manage the time factor better. Our model is only designed for short-term forecasts not for the long-term epidemic change. A deep learning algorithm may be able to deal with this type of forecasting task. Further studies using other algorithms on different forecasting targets, such as the start of a seasonal influenza outbreak and its peak time, are still required to be able to provide more information.

### Conclusions

Our project demonstrated real-time short-term forecasting models on weekly influenza-like illness visits using comprehensive influenza surveillance data. By using an ensemble approach to aggregate the forecasts of 4 machine learning models, we could provide accurate predictions for a 4-week period (nowcast and forecasts for the subsequent 3 weeks) to enhance epidemic preparedness.

## Appendix

Multimedia Appendix 1 The description of data source for the machine learning algorithms.

Multimedia Appendix 2 The feature set and engineering used in machine learning algorithms.

Multimedia Appendix 3 The evaluation metrics for the algorithm performance.

Multimedia Appendix 4 Hyperparameters used in the machine learning models.

Multimedia Appendix 5 The trends of forecasting accuracy with increasing forecasting time horizons by difference evaluation metrics.

Multimedia Appendix 6 Visualization dashboard of the forecasts produced by the five machine learning models on the Fluforecast website.

## Abbreviations

ARIMA: autoregressive integrated moving average model
MAPE: mean absolute percentage error
RMSE: root mean squared error

## References

[1] Yang M, Tan EC, Su J (2017). Cost-effectiveness analysis of quadrivalent versus trivalent influenza vaccine in Taiwan: A lifetime multi-cohort model. Hum Vaccin Immunother.

[2] Yang J, Hsu S, Kuo C, Huang H, Huang T, Wang H, Liu M (2018). An epidemic surge of influenza A(H3N2) virus at the end of the 2016-2017 season in Taiwan with an increased viral genetic heterogeneity. J Clin Virol.

[3] Gong Y, Kuo R, Chen G, Shih S (2018). Centennial review of influenza in Taiwan. Biomedical Journal.

[4] Su C, Tsou T, Chen C, Lin T, Chang S, Influenza Control Group, Infectious Disease Control Advisory Committee (2019). Seasonal influenza prevention and control in Taiwan-Strategies revisited. J Formos Med Assoc.

[5] Jian S, Chen C, Lee C, Liu D (2017). Real-time surveillance of infectious diseases: Taiwan's experience. Health Secur.

[6] Chuang J, Huang AS, Huang W, Liu M, Chou J, Chang F, Chiu W (2012). Nationwide surveillance of influenza during the pandemic (2009–10) and post-pandemic (2010–11) periods in Taiwan. PLoS ONE.

[7] Lipsitch M, Finelli L, Heffernan RT, Leung GM, Redd SC, 2009 H1n1 Surveillance Group (2011). Improving the evidence base for decision making during a pandemic: the example of 2009 influenza A/H1N1. Biosecur Bioterror.

[8] Hillmer SC, Wei WWS (1991). Time series analysis: univariate and multivariate methods. Journal of the American Statistical Association.

[9] Nsoesie EO, Brownstein JS, Ramakrishnan N, Marathe MV (2014). A systematic review of studies on forecasting the dynamics of influenza outbreaks. Influenza Other Respir Viruses.

[10] Paul S, Mgbere O, Arafat R, Yang B, Santos E (2017). Modeling and forecasting influenza-like illness (ILI) in Houston, Texas using three surveillance data capture mechanisms. Online J Public Health Inform.

[11] Xu Q, Gel YR, Ramirez LLR, Nezafati K, Zhang Q, Tsui K (2017). Forecasting influenza in Hong Kong with Google search queries and statistical model fusion. PLoS One.

[12] Feng H, Duan G, Zhang R, Zhang W (2014). Time series analysis of hand-foot-mouth disease hospitalization in Zhengzhou: establishment of forecasting models using climate variables as predictors. PLoS One.

[13] Gharbi M, Quenel P, Gustave J, Cassadou S, La Ruche G, Girdary L, Marrama L (2011). Time series analysis of dengue incidence in Guadeloupe, French West Indies: forecasting models using climate variables as predictors. BMC Infect Dis.

[14] Kane MJ, Price N, Scotch M, Rabinowitz P (2014). Comparison of ARIMA and random forest time series models for prediction of avian influenza H5N1 outbreaks. BMC Bioinformatics.

[15] (2020). Dmlc/Xgboost. GitHub.

[16] Chen S, Xu J, Chen L, Zhang X, Zhang L, Li J (2019). A regularization-based extreme gradient boosting approach in foodborne disease trend forecasting. Stud Health Technol Inform.

[17] Petukhova T, Ojkic D, McEwen B, Deardon R, Poljak Z (2018). Assessment of autoregressive integrated moving average (ARIMA), generalized linear autoregressive moving average (GLARMA), and random forest (RF) time series regression models for predicting influenza A virus frequency in swine in Ontario, Canada. PLoS One.

[18] Bühlmann P, Yu B (2003). Boosting with the L2 Loss. Journal of the American Statistical Association.

[19] Makridakis S, Spiliotis E, Assimakopoulos V (2018). Statistical and Machine Learning forecasting methods: concerns and ways forward. PLoS One.

[20] Smola AJ, Schölkopf B (2004). A tutorial on support vector regression. Statistics and Computing.

[21] Freeze J, Erraguntla M, Verma A (2018). Data Integration and Predictive Analysis System for Disease Prophylaxis: Incorporating Dengue Fever Forecasts.

[22] Ginsberg J, Mohebbi MH, Patel RS, Brammer L, Smolinski MS, Brilliant L (2009). Detecting influenza epidemics using search engine query data. Nature.

[23] Smolinski MS, Crawley AW, Baltrusaitis K, Chunara R, Olsen JM, Wójcik O, Santillana M, Nguyen A, Brownstein JS (2015). Flu Near You: crowdsourced symptom reporting spanning 2 influenza seasons. Am J Public Health.

[24] Broniatowski DA, Paul MJ, Dredze M (2013). National and local influenza surveillance through Twitter: an analysis of the 2012-2013 influenza epidemic. PLoS One.

[25] Santillana M, Nguyen AT, Dredze M, Paul MJ, Nsoesie EO, Brownstein JS (2015). Combining search, social media, and traditional data sources to improve influenza surveillance. PLoS Comput Biol.

[26] Liang F, Guan P, Wu W, Huang D (2018). Forecasting influenza epidemics by integrating internet search queries and traditional surveillance data with the support vector machine regression model in Liaoning, from 2011 to 2015. PeerJ.

[27] Alessa A, Faezipour M (2018). A review of influenza detection and prediction through social networking sites. Theor Biol Med Model.

[28] Poirier C, Lavenu A, Bertaud V, Campillo-Gimenez B, Chazard E, Cuggia M, Bouzillé G (2018). Real time influenza monitoring using hospital big data in combination with machine learning methods: comparison study. JMIR Public Health Surveill.

[29] Ertem Z, Raymond D, Meyers LA (2018). Optimal multi-source forecasting of seasonal influenza. PLoS Comput Biol.

[30] Guo P, Liu T, Zhang Q, Wang L, Xiao J, Zhang Q, Luo G, Li Z, He J, Zhang Y, Ma W (2017). Developing a dengue forecast model using machine learning: A case study in China. PLoS Negl Trop Dis.

[31] Reich NG, McGowan CJ, Yamana TK, Tushar A, Ray EL, Osthus D, Kandula S, Brooks LC, Crawford-Crudell W, Gibson GC, Moore E, Silva R, Biggerstaff M, Johansson MA, Rosenfeld R, Shaman J (2019). Accuracy of real-time multi-model ensemble forecasts for seasonal influenza in the U.S. PLoS Comput Biol.

[32] Kandula S, Yamana T, Pei S, Yang W, Morita H, Shaman J (2018). Evaluation of mechanistic and statistical methods in forecasting influenza-like illness. J R Soc Interface.

[33] Wolpert DH (1992). Stacked generalization. Neural Networks.

[34] Bostock M, Ogievetsky V, Heer J (2011). D³ data-driven documents. IEEE Trans Visual Comput Graphics.

[35] Yang S, Santillana M, Brownstein JS, Gray J, Richardson S, Kou SC (2017). Using electronic health records and internet search information for accurate influenza forecasting. BMC Infect Dis.

[36] Lu FS, Hou S, Baltrusaitis K, Shah M, Leskovec J, Sosic R, Hawkins J, Brownstein J, Conidi G, Gunn J, Gray J, Zink A, Santillana M (2018). Accurate influenza monitoring and forecasting using novel internet data streams: a case study in the Boston metropolis. JMIR Public Health Surveill.

[37] Clothier H, Turner J, Hampson A, Kelly H (2006). Geographic representativeness for sentinel influenza surveillance: implications for routine surveillance and pandemic preparedness. Aust N Z J Public Health.

[38] Polgreen PM, Chen Z, Segre AM, Harris ML, Pentella MA, Rushton G (2009). Optimizing influenza sentinel surveillance at the state level. Am J Epidemiol.

[39] Scarpino SV, Dimitrov NB, Meyers LA (2012). Optimizing provider recruitment for influenza surveillance networks. PLoS Comput Biol.

[40] Greenspan J (2015). Preparing for ILINet 2.0. Online J Public Health Inform.

[41] Woo H, Cho Y, Shim E, Lee J, Lee C, Kim SH (2016). Estimating influenza outbreaks using both search engine query data and social media data in South Korea. J Med Internet Res.

[42] Basile L, Oviedo de la Fuente M, Torner N, Martínez A, Jané M (2018). Real-time predictive seasonal influenza model in Catalonia, Spain. PLoS One.

[43] Reich NG, Brooks LC, Fox SJ, Kandula S, McGowan CJ, Moore E, Osthus D, Ray EL, Tushar A, Yamana TK, Biggerstaff M, Johansson MA, Rosenfeld R, Shaman J (2019). A collaborative multiyear, multimodel assessment of seasonal influenza forecasting in the United States. Proc Natl Acad Sci U S A.

[44] Lu FS, Hattab MW, Clemente CL, Biggerstaff M, Santillana M (2019). Improved state-level influenza nowcasting in the United States leveraging internet-based data and network approaches. Nat Commun.

[45] Yang W, Karspeck A, Shaman J (2014). Comparison of filtering methods for the modeling and retrospective forecasting of influenza epidemics. PLoS Comput Biol.

[46] Yang W, Lipsitch M, Shaman J (2015). Inference of seasonal and pandemic influenza transmission dynamics. Proc Natl Acad Sci U S A.

