# Supplementary material for: Applying Machine Learning Models with An Ensemble Approach for Accurate Real-Time Influenza Forecasting in Taiwan: Development and Validation Study
Source: J Med Internet Res. 2020 Aug 5;22(8):e15394. doi: 10.2196/15394 (PMC7439145; doi:10.2196/15394)
Supplement: Multimedia Appendix 1 [file jmir_v22i8e15394_app1.docx]

**Data description**

**Real-Time Outbreak and Disease Surveillance System.** The Real-Time Outbreak and Disease Surveillance System was established in 2004 [1,2]. This system directly collects the clinical visit data from the electronic medical record system of the emergency department in a hospital. The coverage is higher than 95%. These data are retrieved using information systems of the emergency department every 1–2 h; thus, Taiwan Centers for Disease Control could acquire the data of influenza-like illness visits in the emergency departments in nearly real-time.

The Real-Time Outbreak and Disease Surveillance System database includes the calendar year and week, age group, county, and the weekly number of influenza-like illness visits. It contained 61, 076 records grouped by five age groups and 22 counties from January 1, 2008 to December 16, 2019.

**National Health Insurance Database.** The influenza-like illness visits in outpatient departments were provided by the Nation Health Insurance Administration. The Nation Health Insurance database includes all the healthcare claims data in Taiwan, including the geographical area (country and city) of the hospital visited [2]; International Classification of Diseases, Ninth Revision, clinical modification (ICD-9-CM) diagnostic codes; and age at medical visit. To correctly and quickly acquire reimbursement, healthcare providers intend to submit the data with satisfactory correctness and timeliness. By using the predefined criteria of ICD-9-CM codes, we could select records of influenza-like illness from the Real-Time Outbreak and Disease Surveillance System and the Nation Health Insurance databases with high reliability.

The Nation Health Insurance databases database includes the calendar year and week, type of medical visits (hospitalization or outpatient clinic), age group, county, and the weekly number of influenza-like illness visits. It contained 124,764 records grouped by five age groups and 22 counties from January 1, 2008 to December 16, 2019.

**National Notifiable Disease Surveillance System**. Severe influenza infection with complications is required to be notified in Taiwan. Whenever a patient is diagnosed as having influenza infection with complications and is admitted to the intensive care unit, the details of the patient should be reported to the National Notifiable Disease Surveillance System [2]. The specimens are laboratory-confirmed using a reverse transcriptase polymerase chain reaction (RT-PCR) or viral culture. Therefore, the data from the National Notifiable Disease Surveillance System can be considered a proxy estimate of the number of patients with laboratory-confirmed severe influenza infection.

The National Notifiable Disease Surveillance System database includes the calendar year and week of disease onset, age group, sex, and county of residence. It contained 11,754 records grouped by 19 age groups and 22 counties from January 1, 2008 to December 16, 2019.

All the data aforementioned could be acquired on the open data website of Taiwan CDC with detailed descriptions (https://data.cdc.gov.tw/en/). The data sets are carefully processed before publication without missing values and updated every day. Some descriptive and exploratory data analysis could be found on the website of the Taiwan National Infectious Disease Statistics System (https://nidss.cdc.gov.tw/en/).

**References**

1. Chuang JH, Huang AS, Huang WT, et al. Nationwide surveillance of influenza during the pandemic (2009-10) and post-pandemic (2010-11) periods in Taiwan. PLoS One. 2012;7(4):e36120.
2. Jian SW, Chen CM, Lee CY, Liu DP. Real-Time Surveillance of Infectious Diseases: Taiwan's Experience. Health Secur. 2017;15(2):144–153.
