# Supplementary material for: Applying Machine Learning Models with An Ensemble Approach for Accurate Real-Time Influenza Forecasting in Taiwan: Development and Validation Study
Source: J Med Internet Res. 2020 Aug 5;22(8):e15394. doi: 10.2196/15394 (PMC7439145; doi:10.2196/15394)
Supplement: Multimedia Appendix 2 [file jmir_v22i8e15394_app2.docx]

**Table. The feature sets and the feature engineering used in the machine learning algorithms.**

|  | Features included in the algorithm | | | |
| --- | --- | --- | --- | --- |
|  | **Outpatient visits for influenza-like illness** | **Emergency visits for influenza-like illness** | **Influenza patients with severe complication** | **Others** |
| Data source | National Health Insurance database | Real-Time Outbreak and Disease Surveillance System | National Notifiable Disease Surveillance System |  |
| ARIMA^a^ | Weekly counts | Weekly counts  Proportion of influenza-like illness | Weekly counts | Weekends and holidays  Lunar New Year |
| RF^b^ | Weekly counts with lag  Moving average  Moving difference  Proportion of influenza-like illness | Weekly counts with lag  Moving average  Moving difference  Proportion of influenza-like illness |  | Weekends and holidays  Lunar New Year |
| SVR^c^ | Weekly counts with lag  Moving average  Moving difference  Proportion of influenza-like illness | Weekly counts with lag  Moving average  Moving difference  Proportion of influenza-like illness | Weekly counts with lag  Moving average  Moving difference | Weekends and holidays  Lunar New Year |
| XGB^d^ | Weekly counts with lag  Moving average  Moving difference  Proportion of influenza-like illness | Weekly counts with lag  Moving average  Moving difference  Proportion of influenza-like illness | Weekly counts with lag  Moving average  Moving difference | Weekends and holidays  Lunar New Year |

^a^ARIMA, autoregressive integrated moving average;

^b^RF, random forest;

^c^SVR, support vector regression;

^d^XGB, extreme gradient boosting;

**Interpretation of selected features**

1. Holiday event: the influenza-like illness visits at emergency departments considerably increase during the holidays when outpatient service is closed and sometimes patients crowd the emergency departments.
2. Moving average (MA): Moving average shows the average of influenza-like illness visits. It is more smooth and may describe the trends of influenza-like illness visits better.
3. Moving difference (MD): MD1 is the difference between lag1-lag2 data, MD2 is the difference bwteen lag2-lag3 data, and so on. The moving difference of two adjacent lag suggests the variation of influenza-like illness visits. Positive and large moving differences in continuous lags suggest rapid increases resulting a potential outbreak.
4. Proportion of influenza-like illness: influenza-like illness visits divided by total visits is an scae-free indicator of influenza-like illness visits.
