# Supplementary material for: Applying Machine Learning Models with An Ensemble Approach for Accurate Real-Time Influenza Forecasting in Taiwan: Development and Validation Study
Source: J Med Internet Res. 2020 Aug 5;22(8):e15394. doi: 10.2196/15394 (PMC7439145; doi:10.2196/15394)
Supplement: Multimedia Appendix 3 [file jmir_v22i8e15394_app3.docx]

**Evaluation Metrics**

The metrics we used to evaluate the model performance included Pearson’s correlation, root mean squared error, mean absolute percentage error, and hit rate. Let $y$ be the real observed value and $\hat{y}$ be the forecasting value, RMSE, MAPE and hit rate were calculated using the following equations:

- Root mean squared error: $\sqrt{\frac{1}{N}\sum_{i=1}^{N} {(y_{i}-\hat{y}_{i})}^{2}}$
- Mean absolute percentage error: $\frac{1}{N}\sum_{i=1}^{N} \frac{\left|y_{i}-\hat{y}_{i}\right|}{y_{i}}.$
- Hit rate: $\frac{1}{N}\sum_{i=1}^{N} I(sign(\hat{y}_{i}-\hat{y}_{i-1})-sign(y_{i}-y_{i-1})),$where the indicator function $I\left(x\right)$ is 1 if x is equal to 0.
