# Supplementary material for: Applying Machine Learning Models with An Ensemble Approach for Accurate Real-Time Influenza Forecasting in Taiwan: Development and Validation Study
Source: J Med Internet Res. 2020 Aug 5;22(8):e15394. doi: 10.2196/15394 (PMC7439145; doi:10.2196/15394)
Supplement: Multimedia Appendix 4 [file jmir_v22i8e15394_app4.docx]

**Table. Hyperparameters used in the machine learning models.**

| **Model** | **R package** | **function** | **parameters** |
| --- | --- | --- | --- |
| ARIMA | forecast | Auto.arima | Stepwise=False  Seasonal=True  Non-seasonal order   - p: 2 to 5 - d: 0, 1, 2 - q: 2 to 5   Seasonal order   - P: 1, 2 - D: 1, 2 - Q: 1, 2 |
| SVR^a^ | e1071 | svm | Kernel=”linear”  Type=”eps-regression”  Cost=1  Epsilon=0.1 |
| RF | RandomForest | randomForest | Mtry=tuneRF(p/3) ^b^  NTree=500 |
| XGB | xbgboost | Xgboost^c^ | Nrounds=500  Max_depth=10  Eta=0.3  Colsample_bytree=0.5  Subsample=0.7 |

^a^Used both in individual model and ensemble model.

^b^p indicates number of predictors.

^c^Some parameter names in xgboost() possibly are changed in new xbgboost release.

ARIMA: autoregressive integrated moving average.

RF: random forests.

SVR: support vector regression.

XGB: extreme gradient boosting.
