# Supplementary figures and images for: Applying Machine Learning Models with An Ensemble Approach for Accurate Real-Time Influenza Forecasting in Taiwan: Development and Validation Study
Source: J Med Internet Res. 2020 Aug 5;22(8):e15394. doi: 10.2196/15394 (PMC7439145; doi:10.2196/15394)

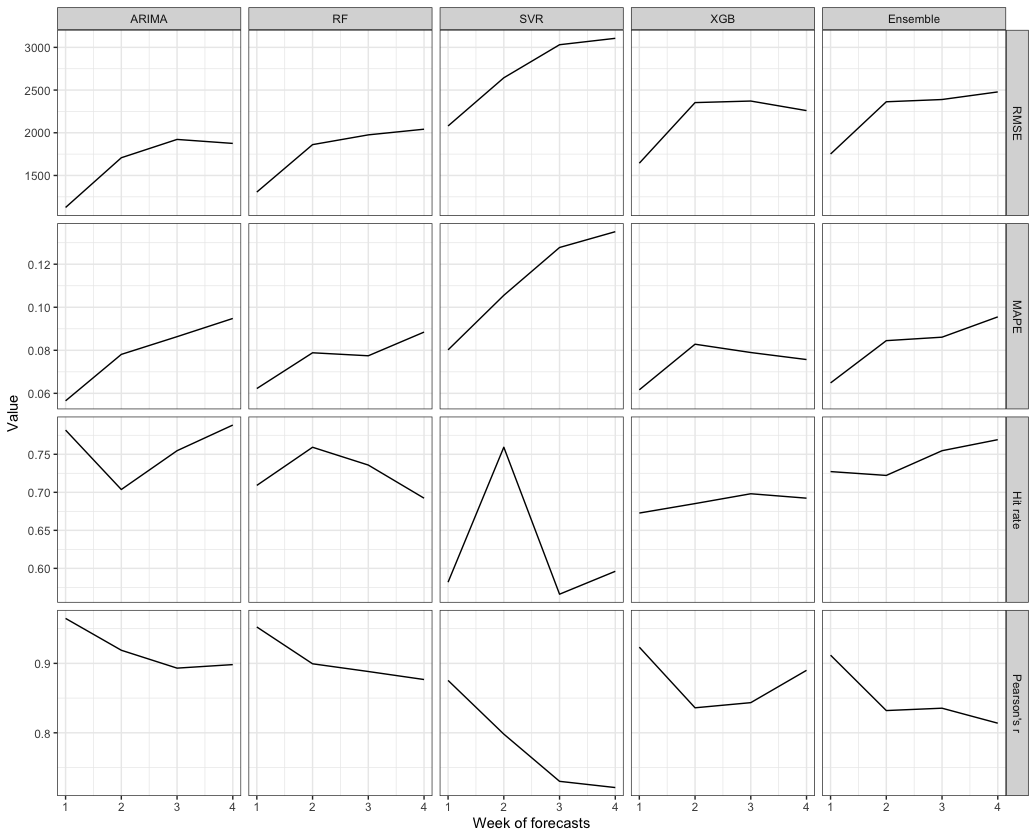

Supplement: Multimedia Appendix 5 [file jmir_v22i8e15394_app5.png]

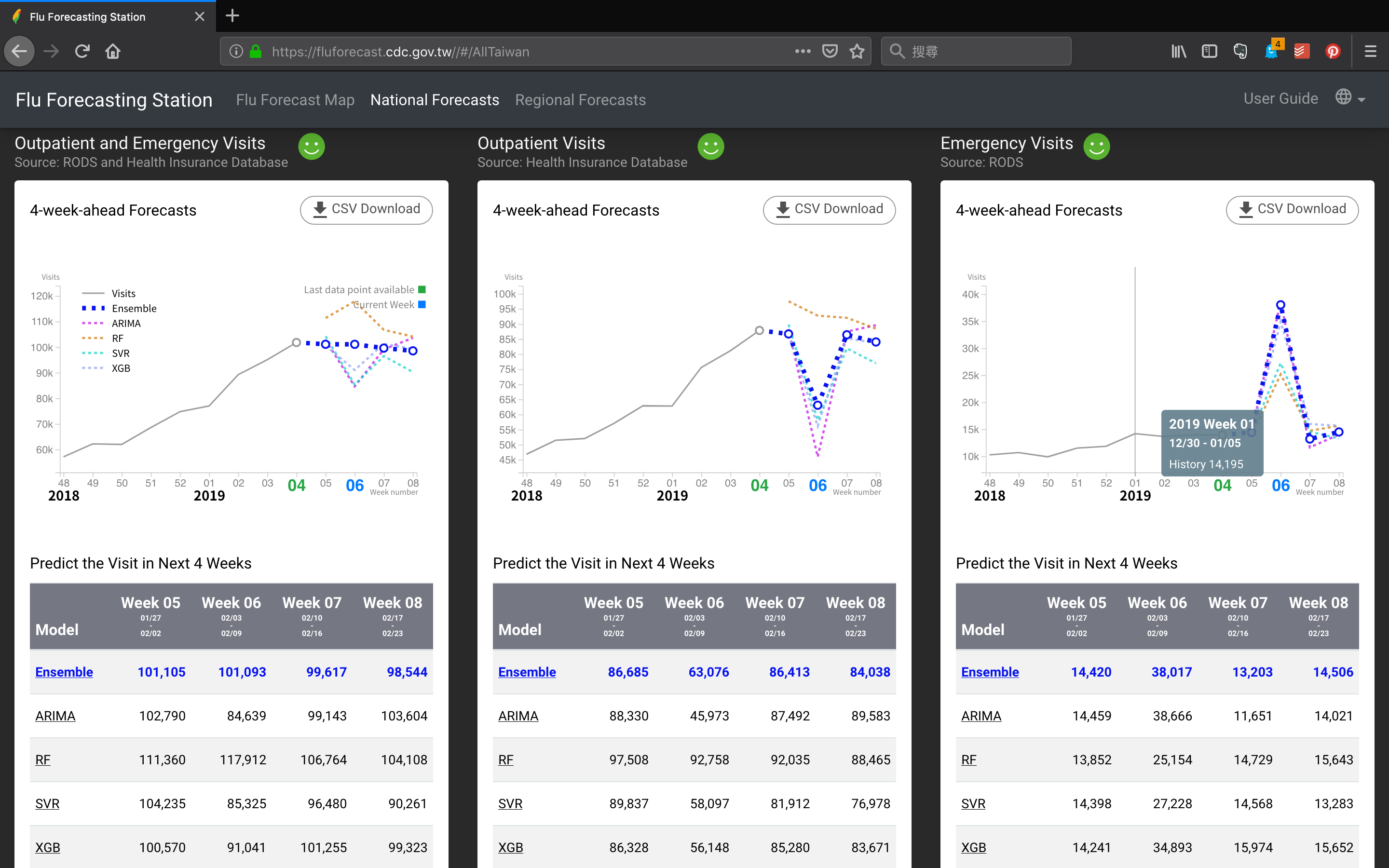

Supplement: Multimedia Appendix 6 [file jmir_v22i8e15394_app6.png]
